# Rapid “Breath-Print” of Liver Cirrhosis by Proton Transfer Reaction Time-of-Flight Mass Spectrometry. A Pilot Study.

**DOI:** 10.1371/journal.pone.0059658

**Published:** 2013-04-03

**Authors:** Filomena Morisco, Eugenio Aprea, Vincenzo Lembo, Vincenzo Fogliano, Paola Vitaglione, Giovanna Mazzone, Luca Cappellin, Flavia Gasperi, Stefania Masone, Giovanni Domenico De Palma, Riccardo Marmo, Nicola Caporaso, Franco Biasioli

**Affiliations:** 1 Department of Clinical Medicine and Surgery, University of Naples “Federico II”, Naples, Italy; 2 Department of Agriculture, University of Naples “Federico II”, Portici (Naples), Italy; 3 Food Quality and Nutrition Department, IASMA Research and Innovation Centre, S. Michele a/A (Trento), Italy; 4 Gastroenterology Unit, Polla Hospital, Polla (Salerno), Italy; Medical University Graz, Austria

## Abstract

The aim of the present work was to test the potential of Proton Transfer Reaction Time-of-Flight Mass Spectrometry (PTR-ToF-MS) in the diagnosis of liver cirrhosis and the assessment of disease severity by direct analysis of exhaled breath. Twenty-six volunteers have been enrolled in this study: 12 patients (M/F 8/4, mean age 70.5 years, min-max 42–80 years) with liver cirrhosis of different etiologies and at different severity of disease and 14 healthy subjects (M/F 5/9, mean age 52.3 years, min-max 35–77 years). Real time breath analysis was performed on fasting subjects using a buffered end-tidal on-line sampler directly coupled to a PTR-ToF-MS. Twelve volatile organic compounds (VOCs) resulted significantly differently in cirrhotic patients (CP) compared to healthy controls (CTRL): four ketones (2-butanone, 2- or 3- pentanone, C8-ketone, C9-ketone), two terpenes (monoterpene, monoterpene related), four sulphur or nitrogen compounds (sulfoxide-compound, S-compound, NS-compound, N-compound) and two alcohols (heptadienol, methanol). Seven VOCs (2-butanone, C8-ketone, a monoterpene, 2,4-heptadienol and three compounds containing N, S or NS) resulted significantly differently in compensate cirrhotic patients (Child-Pugh A; CP-A) and decompensated cirrhotic subjects (Child-Pugh B+C; CP-B+C). ROC (Receiver Operating Characteristic) analysis was performed considering three contrast groups: CP *vs* CTRL, CP-A *vs* CTRL and CP-A *vs* CP-B+C. In these comparisons monoterpene and N-compound showed the best diagnostic performance.

**Conclusions:**

Breath analysis by PTR-ToF-MS was able to distinguish cirrhotic patients from healthy subjects and to discriminate those with well compensated liver disease from those at more advanced severity stage. A breath-print of liver cirrhosis was assessed for the first time.

## Introduction

Smelling the exhaled breath of patient is an ancient approach of expert clinicians to recognize some illnesses since the times of Hippocrates, who first described *fetor oris* and *fetor hepaticus* in his treatise on breath odour and disease. In particular, the sweet smell of acetone in human breath is associated to uncontrolled diabetes, while the fishy and urine-like smells are due to liver disease and kidney failure, respectively [1]–[3].

Liver plays a key role in metabolism and, even in the early stages of chronic liver damage, a metabolic impairment can be usually evidenced leading to the over-production of various endogenous compounds which concentrate in the blood and, if volatiles, are present in the exhaled breath. This is the premise to perform a non invasive diagnosis with breath analysis [4]. However, only in the last few decades this approach was made possible due to the development of a sensitive gas-chromatographic and mass-spectrometric instruments able to measure Volatile Organic Compounds (VOCs) with sufficient accuracy and sensitivity [5].

Up to now, the clinical utility of breath analysis was evaluated for different diseases as, for instance, the monitoring of diabetes mellitus and in the screening for lung and colorectal cancer [1], [6], [7]. Very few information are available about its possible use in patients with liver cirrhosis [1].

Among the different methods for breath analysis, direct injection mass spectrometry [8] has many advantages as previously highlighted [9]. Summarizing: it is a completely non-invasive approach; it does not need the administration of drugs or marker compounds, as in the classical “breath test” [10]; it can be performed in real-time without breath sample pre treatment. A particular advantage of our method, in comparison to other recent approaches in gastrointestinal disease [7], is the immediate availability of the results at the time of the sampling, the easy application, the low risk of artifacts and the abolition of procedural steps related to the filling of bag, the adsorption of VOCs on to sorbent cartridge, the desorption of VOCs and finally the possibility to separate the breath inhaled fraction (environmental contaminants) from the end-tidal breath.

Proton Transfer Reaction Mass Spectrometry (PTR-MS) [11] is a particular implementation of direct injection mass spectrometry characterized by very low detection limits and by a soft chemical ionization often producing the molecular ion only. While most implementation of PTR-MS so far were based on a quadrupole mass analyzer, recently a new version implementing a Time-of-Flight mass analyzer has been realized (PTR-ToF-MS) [12]. This new version is characterized by a wider mass range and a better time resolution (one spectrum in a split second), respect to the previous ones. Moreover the good mass resolution and accuracy allowing molecular formula identification. PTR-ToF-MS has been recently applied for breath analysis of humans [13] and animal models [14].

This work aimed to evaluate whether breath analysis by PTR-ToF-MS can be used for a rapid, direct and non invasive diagnosis of liver cirrhosis, as well as for the assessment of disease severity.

## Subjects and Methods

### Subjects and Treatment

The study protocol was approved by the Ethical Committee of the University of Naples “Federico II” and all participants signed the informed consent before the enrolment. Twelve patients (M/F 8/4, mean age 70.5, 42–80 years) with liver cirrhosis of different etiology and status and 14 healthy subjects (M/F 5/9, mean age 52.3, 35–77 years) were enrolled in the study. The principal characteristics of patients and controls are reported in **Table 1**. All subjects were Caucasian. The diagnosis in patients with cirrhosis was previously formulated on the basis of clinical ultrasonographic and biochemical parameters. The etiology of cirrhosis was viral in 9 patients (8 HCV and 1 HBV) and metabolic in 3 patients. The Child-Pugh class of subjects in the patients group was A in 6, B in 3 and C class in 3.

**Table 1:** Characteristics of studied subjects.

*Variable*	*Cirrhotic (n = 12)*	*Controls (n = 14)*	*p value*
**Age** (years): mean±SD	70.5 9.8	52.3 13.7	0.006
**Gender**			
Male: subject numbers (%)	8 (66.7%)	5 (35.7%)	ns
Female: subject numbers (%)	4 (33.3%)	9 (64.3%)	ns
**BMI**: mean±SD	27.2 3.5	26.5 4.3	ns
**Smoker**			
Yes: subject numbers (%)	1 (8.3%)	3 (21.4%)	ns
No: subject numbers (%)	11 (91.7%)	11 (78.6%)	ns
**Serum bilirubin** (mg/dL): mean±SD	1.4±0.6	0.7±0.3	–
**Serum albumin** (g/L): mean±SD	3.7±0.3	4.1±0.3	–
**INR** (ratio): mean±SD	1.4±0.4	1.0±0.1	–
**ALT** (times ULN): mean±SD	1.2±0.7	0.52±0.2	–
**Platelets** (x109/L): mean±SD	114±60	287±37	–
**Alpha-FP** (ng/mL): mean±SD	8.4±11	–	–
**Child-Pugh score**			
Class A: subject numbers (%)	6 (50.0%)		–
*Etiology:* HCV, HBV, Cryptogenetic	5/0/1	–	–
Class B: subject numbers (%)	3 (25.0%)		–
*Etiology:* HCV, HBV, Cryptogenetic	3/0/0	–	–
Class C: subject numbers (%)	3 (25.0%)	–	–
*Etiology:* HCV, HBV, Cryptogenetic	0/1/2		–

**Abbreviations:** INR, International Normalized Ratio; ALT, alanine transaminase; Alpha-FP, Alpha-Fetoprotein; HCV, Hepatitis C Virus; HBV, Hepatitis B Virus.

The Child-Pugh is a score routinely used in hepatology to assess stage and prognosis of cirrhosis; it is based on functional tests (bilirubin, INR or prothrombin activity, albumin) and two clinical parameters: Portal-Systemic Encephalopathy (EPS) and ascites. The Child-Pugh score can range from class A (well compensated liver cirrhosis) to class C (end stage cirrhosis). No patients had a history of surgical shunt or transjugular intrahepatic portosystemic shunt, severe chronic cholestasis, diabetes mellitus, neoplasia, kidney failure, or recent weight reduction.

Breath sampling was carried out in the morning from fasting subjects. Participants were also asked to refrain, since the evening before the measurement, from smoking, chewing gum, using mouthwash, brushing teeth, drinking alcohol and coffee and consuming foods containing garlic, onion, mint and similar flavored meals. To this purpose a standardized dinner was consumed the evening before the sampling including a serving of fish or white meat, steamed vegetables, white bread, apple or pear. Physical exercises were also avoided over the 24 hours before measurements. Participants in the study were subjected to a dietary questionnaire to assess their eating habits and food eaten in the days before the measurement of breath. Each subject was asked to indicate the average portion and the frequency of intake of over 60 foods belonging to the following groups: milk and dairy products, fish, meat and eggs, meats, cereals and cereal products, fruits and vegetables, snacks and soft drinks and alcohol. The food questionnaires were compiled with the help of photographs and images to calculate the average portion taken. The food questionnaires were drawn up subsequently by a software that can make a semi-quantitative assessment of the diet.

### Breath Sampling and PTR-ToF-MS

Real time breath analysis was performed using a buffered end-tidal (BET) on-line sampler [15] coupled to a Proton Transfer Reaction Time-of-Flight Mass Spectrometer (PTR-ToF-MS, Ionicon Analytik - Austria). Subject is sitting in front of the interface and asked to breath normally room air. After a short time, the operator asks the subject to give a single exhalation in a disposable mouthpiece, provided with a sputum trap, connected to the BET system. The BET system allows the collection of the last 40 ml of exhaled breath gas known as end-tidal fraction. This fraction is the richest in those molecules derived from exchange at the alveolar-capillary membrane and less affected by inhaled breath air gas. Furthermore the use of BET system avoids the effect of hyperventilation on volatile concentration. The fraction of exhaled gas collected through the BET system is drawn directly to the drift tube of a PTR-ToF-MS used as on-line detection and recording system of the volatile organic compounds spectra.

### Data Analysis and Statistics

Spectra were acquired using the data acquisition software TOF-DAQ (Tofwerk AG, Switzerland) with a mass range of 10–400 Th and stored in HDF5 format for efficient data storage and direct access to data structure and considered for data analysis.

Signal distortions caused by the detector dead time were corrected before mass calibration, peak detection and area extraction, which were performed according to the procedure described in [16] using a cumulative peak fitting [17]. Internal calibration was based on three peaks always present in the PTR-MS spectra at m/z = 21.0221 (H_3_^18^O^+^), 29.9974 (NO^+^) and 59.0491 (protonated acetone: C_3_H_7_O^+^). Throughout the article, we use 3 decimal figures for estimated m/z values and 4 for the expected exact ones.

Peak intensity in part per billion (ppbv) was estimated by the formula described in Lindinger et al. [18] using a constant value for the reaction rate constant (k = 2.10^−9^ cm^3^ s^−1^). This introduces a systematic error for the absolute concentration for each compound that is in most cases below 30% and could be accounted for if the actual rate constant is available [19].

Exploratory examinations of the clinical data involved the calculation of descriptive statistics (as appropriate, the mean, median, standard deviation (SD), proportion and 95% confidence interval were computed). Continuous quantitative breath data, being not normally distributed, were summarized with their median and median absolute deviation. Comparison of continuous variables was performed with Mann–Whitney U test analysis. A significant level of *p*<0.05 was chosen. A typical PTR-ToF-MS spectrum contains hundreds of peaks even in the case of breath analysis. This has been considered in the case of multiple comparison by applying the false discovery rate control [20].

To highlight possible relationship between VOCs and biochemical parameters in patients with cirrhosis the Pearson’s correlation was measured. Not normally distributed variables were transformed according to the Box-Cox method [21].

ROC (Receiver Operating Characteristic) curves were used to calculate the performance of diagnostic procedures and for calculating the best point of separation between sensitivity and specificity of each of them. Sensitivity and specificity were calculated according to Sackett [22]. Given the small sample size and to reduce the possible beta error, a *p value* lower than 0.10 was considered significant and clinically valuable. Data were analysed using the Statistica 9.1 (StatSoft, USA) software.

## Results

### VOCs Identification

The analysis of the acquired spectra allowed the extraction of 285 mass peaks 51 of them being significantly different (*p*<0.05) in cirrhotic patients (CP) compared to healthy controls (CTRL). The false discovery rate method has been used to take into account the multiple comparisons and provided a selection of twenty-six peaks, related to 12 compounds. The list of measured and theoretical monoisotopic masses detected, the corresponding mass errors, the corresponding VOC identified by the sum formula of each monoisotopic peak and, in some cases, by fragmentation comparison, are reported in **Table 2** (**Figure S1**).

**Table 2:** List of the 12 peaks considered in this study.

*Measured mass (Th)*		*Theoretical mass (Th)*	*Error (ppm)* *	*Tentative identification*	*Sum formula of base peak*
33.033	33.0335		−10.7	Methanol	CH4O·H+
73.065		73.0648	8.1	2-butanone	C4H8OH+
87.082		87.0804	18.4	2- or 3-pentanone	C5H10OH+
89.030		89.0294	9.8	NS-compound	C3H7NS+
91.030		91.0291	7.4	N-compound	C5H3N2+
95.086		95.0855	4.3	Heptadienol	C7H11+
121.033		121.0318	10.8	S-compound	C4H8O2S·H+
129.126		129.1274	−14.4	C8-ketone	C8H16O·H+
135.119		135.1168	17.5	Terpene related	C10H14·H+
137.137		137.1325	35.2	Monoterpenes	C10H17+
143.144		143.1430	6.7	C9-ketone	C9H18O·H+
149.098		149.0995	−6.5	Sulfoxide-compound	C7H16OS·H+

* The difference between measured and expected mass is reported as part per million (ppm).

The identified VOCs could be grouped in the chemical classes of alcohol (heptanedienol and methanol), ketons (2-butanone, 2- or 3-pentanone, and other two VOCs, most probably 2-octanone, i.e. C8-ketone; and 2-nonanone, i.e. C9-ketone), terpenes (monoterpene tentatively identified as limonene, and a terpene related compound tentatively identified as p-cymene), sulphur and nitrogen (Sulfoxide compound, S-compound, NS- and N-compound) compounds.

### VOCs Quantification

**Table 3** shows the median concentration of the identified VOCs in the whole group of cirrhotic patients (CP) and in healthy controls (CTRL) as well as in the subgroups of CP classified as Child-Pugh A (CP-A) and Child-Pugh B+C (CP-B+C). No difference in the spectrum of VOCs has been observed in relation to age. All, but S-compound, have a higher concentration in CP breath than in CTRL one. Further analysis on patient subgroups showed that no difference in VOC concentration was in CP-A *vs* CTRL, but for N-compound.

**Table 3:** Markers significantly different between CP *vs* CTRL, CP-A *vs* CTRL and CP-A *vs* CP-B+C.

*VOC*	*Concentration (ppb_v_)*		*p value*	*Concentration (ppb_v_)*		*p value*	*Concentration (ppb_v_)*		*p value*
	*Median and Median absolute deviation*			*Median and Median absolute deviation*			*Median and Median absolute deviation*		
	CP	CTRL		CP-A	CTRL		CP-A	CP-B+C	
**Ketones**									
2-butanone	3.2±0.5	2.6±0.5	0.027	3.1±0.1	2.6±0.5	ns	3.1±0.1	4±1	0.041
2- or 3-pentanone	1.4±0.3	1.06±0.16	0.020	1.0±0.2	1.06±0.16	ns	1.0±0.2	1.5±0.4	ns
C8-ketone	0.13±0.02	0.09±0.01	0.005	0.11±0.01	0.09±0.01	ns	0.11±0.01	0.19±0.08	0.009
C9-ketone	0.10±0.02	0.07±0.02	0.027	0.11±0.02	0.07±0.02	ns	0.11±0.02	0.09±0.08	ns
**Terpenes**									
Monoterpenes	6.7±5	1.3±0.4	0.000	3±1	1.3±0.4	ns	3±1	54±52	0.002
Terpene related	0.6±0.1	0.38±0.03	0.006	0.54±0.06	0.38±0.03	ns	0.54±0.06	0.8±0.3	ns
**S and N containing compounds**									
Sulfoxide-compound	0.08±0.02	0.06±0.02	0.027	0.02±0.03	0.06±0.02	ns	0.02±0.03	0.10±0.03	ns
S-compound	0.09±0.03	0.13±0.03	0.011	0.10±0.02	0.13±0.03	ns	0.10±0.02	0.06±0.04	0.041
NS-compound	0.86±0.26	0.58±0.20	0.046	0.6±0.2	0.58±0.20	ns	0.6±0.2	1.4±0.7	0.004
N-compound	0.40±0.07	0.16±0.06	0.020	0.5±0.2	0.16±0.06	0.002	0.5±0.2	0.2±0.3	0.015
**Alcohol**									
Methanol	528±218	279±134	0.041	404±128	279±134	0.034	404±128	725±320	ns
Heptadienol	2.5±1.4	0.9±0.2	0.020	1.9±0.5	0.9±0.2	ns	1.9±0.5	6±4	0.002

**Abbreviations:** CP, cirrhotic patients; CTRL, healthy controls; CP-A, cirrhotic Child Pugh A; CP-B+C, cirrhotic Child Pugh B+C.

Otherwise, seven VOCs had a different concentration in CP-A *vs* CP-B+C; specifically, five VOCs were at higher concentration (2-butanone, C8-ketone, monoterpene, NS-compound, heptadienol) and two were at lower concentration (S-compound, N-compound) in the breath of CP-B+C compared to CP-A. Eleven compounds (all but N-compound) showed significantly different concentration in CTRL *vs* CP-B+C (**Table S1**).

### VOCs Correlation with Liver Function Test

**Figure 1:**
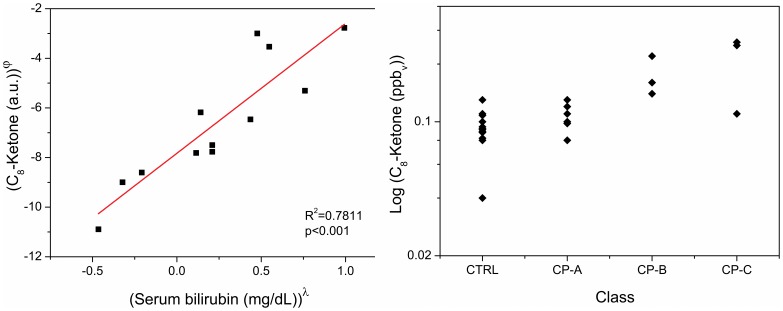
Correlation and distribution of the C8-ketone. Correlation between serum bilirubin and C8-Ketone (**panel a**) and distribution of the C8-ketone breath’s concentrations in healthy controls (CTRL) and the 3 classes of cirrhotic patients (CP) (**panel b**). Variable in **panel a** are power transformation of original values (λ = 0.1152; φ = −0.9871).

As reported in **Table 4** significant correlation between the 12 identified VOCs and biochemical parameters of liver function was found. Serum bilirubin showed a positive correlation with 6 VOCs: monoterpene, methanol, 2-butanone, heptadienol, C8-ketone, terpene related. The highest correlation was found for the C8-ketone. **Figure 1** shows the correlation between serum bilirubin and C8-Ketone (**panel a**) and the distribution of the C8-ketone breath concentrations in CTRL and the 3 classes of CP (**panel b**). Prothrombin activity is negatively correlated to the monoterpene and the C8-ketone. These correlations were also confirmed by Spearman’s Rank Correlation Coefficient (data not shown) [23]. No significant correlation was found between serum albumin level and the 12 VOCs identified.

**Table 4:** Pearson correlation between VOCs and biochemical parameters.

*VOCs*	*Serum bilirubin*		*Serum albumin*		*Prothrombin activity*	
	r	p	r	p	r	p
**Ketones**						
2-butanone	**0.733**	**0.007**	−0.187	0.560	−0.412	0.183
2- or 3-pentanone	0.531	0.075	−0.095	0.769	−0.368	0.239
C8-ketone	**0.895**	**<0.001**	−0.172	0.593	**−0.642**	**0.024**
C9-ketone	−0.106	0.743	0.213	0.506	0.033	0.919
**Terpenes**						
Monoterpenes	**0.693**	**0.012**	−0.164	0.610	**−0.592**	**0.042**
Terpene related	**0.635**	**0.026**	−0.089	0.784	−0.407	0.190
**S and N containing compounds**						
Sulfoxide-compound	0.205	0.522	0.053	0.870	−0.013	0.968
S-compound	−0.432	0.161	0.010	0.976	0.106	0.742
NS-compound	0.558	0.06	0.042	0.897	−0.298	0.347
N-compound	−0.510	0.091	0.411	0.184	0.499	0.099
**Alcohol**						
Methanol	**0.578**	**0.045**	−0.210	0.512	−0.350	0.265
Heptadienol	**0.618**	**0.032**	−0.060	0.853	−0.540	0.070

In bold significant correlation (*p*<0.05).

### ROC Analysis

To evaluate whether individual VOCs or an appropriate combination of them can discriminate among groups, ROC analysis was performed.

The performance of a test to separate patients with (sensitivity) and without (specificity) a specific disease is graphically expressed by the ROC curve. The area under the curve allows a comparison of the diagnostic performance of different tests: the greater is the area under the curve, the better is the ability to separate the two groups of patients.

Three contrast groups were considered, i.e. CP *vs* CTRL, CP-A *vs* CTRL and CP-A *vs* CP-B+C. The area under curve (AUC) of VOCs with the highest diagnostic performance (*p* value<0.10) and the coordinate of the ROC curve with the best value able to separate the compared groups are summarized in **Table 5**. Given the higher p-value used in this analysis we considered also dimethyl sulphide that did not meet the false discovery rate criterion.

**Table 5:** ROC curve analysis of detected markers.

*Comparison*	*Marker*	*AUC*	*P* a	*Best value of separation*	*Sensitivity %*	*Specificity %*
**CP vs CTRL**						
	2-butanone	.756	.027	2.90	75	79
	2- or 3-pentanone	.768	.021	1.08	75	64
	C8-ketone	.815	.006	.10	83	64
	C9-ketone	.756	.027			
	Monoterpene	.887	.001	2.16	83	86
	Terpene related	.810	.007	.39	83	64
	S-compound	.208	.012	.11	83	72
	Sulfoxide-compound	.756	.027			
	N-compound	.768	.021	.19	83	64
	Heptadienol	.768	.021	1.48	83	72
	Methanol	.738	.040	485.73	58	86
**CP-A vs CTRL**						
	C9-ketone	.786	.048	.099	67	99.7
	Monoterpene	.774	.058	2.16	66	96
	N-compound	.929	.003	.26	83	94
	Dimethyl sulphide	.750	.083	6.28	83	64
**CP-A vs CP-B+C**						
	2-butanone	.139	.037	178.5	83	99
	2- or 3-pentanone	.167	.055	1.13	1	67
	C8-ketone	.056	.010	.11	1	67
	Monoterpene	.000	.004	6.7	1	99
	S-compound	.861	.037	0.04	83	99
	NS-compound	.028	.006	.85	1	83
	N-compound	.917	.016	.14	83	99
	Heptadienol	.000	.004	2.30	1	83

a Null hypothesis: true area = 0.5.

**Figure 2:**
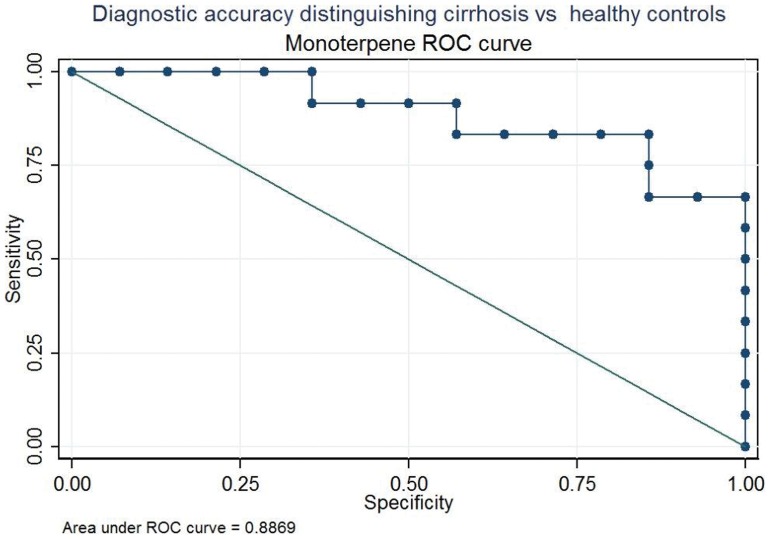
Receiving operating characteristic (ROC) curve for Monoterpene. Diagnostic accuracy distinguishing cirrhotic patients (CP) and healthy controls (CTRL).

Eleven VOCs (all but NS-compound), have a good diagnostic performance to discriminate CP *vs* CTRL. In this comparison the monoterpene related peak was the one with the highest diagnostic performance. Using the best cut-off of separation (2.16 ppbv) the sensitivity and specificity were respectively of 83% and 86%, as shown in **Figure 2**.

Four VOCs (C9-ketone, monoterpene, N-compound, dimethyl sulphide) have a good diagnostic performance to discriminate CP-A *vs* CTRL. In this comparison the N-compound was the VOC with the highest diagnostic performance. Using the best cut-off of separation (0.26 ppbv) the sensitivity and specificity were respectively of 83% and 86%.

Eight peaks (2-butanone, 2- or 3-pentanone, C8-ketone, monoterpene, S-compound, NS-compound, N-compound, heptadienol) have a good diagnostic performance to discriminate CP-A *vs* CP-B+C. In this comparison again the monoterpene related peak was the one with the highest diagnostic performance. Using the best cut-off of separation (6.7 ppbv) the sensitivity and specificity were always 100%.

## Discussion

In this study PTR-ToF-MS was used for the first time to analyze exhaled breath of patients with liver cirrhosis aiming at verifying its applicability as non-invasive tool for diagnosis of cirrhosis.

Twelve different VOCs, including ketones (2-butanone, 2-or 3-pentanone, C8-ketone, C9-ketone), terpenes (monoterpene, terpene related), S and N containing compounds (Sulfoxide-compound, S-compound, NS-compound, N-compound) and alcohols (heptadienol, methanol) were significantly different between cirrhotic and healthy subjects.

The only previous work on this topic, investigating molecules responsible of *fetor hepaticus* [1] examined the breath of cirrhotic patients by GC-MS combined with thermal desorption. Authors identified four VOCs (three ketones: acetone, 2 pentanone, 2-butanone and one sulphur compound: dimethyl sulphide) being at higher concentration in cirrhotics’ breath than in controls’ one [1]. Interestingly, the chemical classes of discriminating VOCs found in our study (ketones and sulphur compounds) were the same, and the PTR-ToF-MS attained a more complete picture of the breath compounds also allowing to distinguish patients according to disease severity. Seven VOCs have different concentrations among groups being significantly more (2-butanone, C8-ketone, monoterpene, NS-compound and heptadienol) and less (S-compound and N-compound) abundant in patients with advanced disease (Child B and C patients) compared to those with compensated cirrhosis (Child A patients). Although in this last comparison the small sample did not allow definitive statements, however, we have reported the result as the trend was preserved.

The increased concentration of **ketones** in exhaled breath of patients with advanced cirrhosis might be dependent from increased insulin resistance and from a different metabolic response to fasting in patients with advanced cirrhosis *vs* those with compensated disease [22]. In fact, insulin resistance, that usually increase in patients with end stage liver disease [24], [25], favored the lipolysis and free fatty acids β-oxidation led to the formation of ketones [26]. This hypothesis is further supported by the direct correlation between the levels of C8-ketone, present in the breath, and serum levels of bilirubin, as well as with the Child –Pugh stage of cirrhosis. On the other hand, the hypothesis that response to fasting might have a role in discriminating breath composition depending on liver disease severity is also consistent with data reported by Van der Velde and co-workers [1] who analyzed breath of subjects 30 minutes from food intake (a time that might be too short to modify concentration of ketones from previous fasting) and with the observation by Mathews and co-workers [27] that a reduced of CYP2E1 enzyme activity (as in liver disease) increased breath ketones in rats.

The peak at m/z 137.137, is a terpene-related peak tentatively identified as limonene. It was 15 folds more abundant in CP than in CTRL and ROC analysis even assigned to this feature a prognostic significance for liver disease. This evidence can be explained by the diet composition or by the lacking efficacy of liver metabolism leading to a higher concentration of **terpenes** in cirrhotic patients than in healthy subjects.

In a previous work, the high concentration of limonene in the lung air of 37% (9 out 24) of patients with liver disease was suggested to be dependent from the frequency of fruit juice consumption [28]. However, this possibility was ruled out in the present study since breath limonene did not correlate with citrus product consumption (as recorded by a food frequency questionnaire relative to the week before breath sampling). Moreover, none of the drugs used by subjects could originate terpenes neither directly or indirectly by affecting isoprenoid biosynthetic pathway. Since metabolism of limonene includes a first step in the liver [29], where it may be transformed in carveol metabolites or perillyl metabolites by CYP2C enzymes [30], it has been hypothesized that a deficient liver metabolism, in end-stage disease, may determine a reduction of limonene biotransformation and its accumulation in the original form with a consequent retard of excretion and a high abundance in the exhaled breath.

The increased concentration of some **sulphur containing compounds**, in CPs’ breath was consistent with the well known incomplete metabolism of sulphur containing amino acids typical of liver disease [1], [31], [32].

The production of various nitrogen species increases during oxidative stress and **nitrogen compounds** are considered a good markers of oxidative damage [33]. In liver injury, the concentration of nitrogen compounds, such as ammonia, increased in the blood when the removal of ammonia through the conversion to urea is limited due to the impairment of liver function [34].

The increased **methanol** in human breath was already observed by other authors and it was related to pectin degradation and explained with a different amount of fruit intake by cirrhotic patients [35]. However, also in this case the dietary intake analysis demonstrated no differences in fruit consumption between CTRL and CP, thus the different methanol breath amounts in the two groups, might be due to other reasons. The imbalance of microflora composition found in cirrhotic patients [36], could account for a different colon fermentation activity and, in turn, for the different concentration of methanol in the breath.

Furthermore, some VOCs, such as monoterpene and C8-ketone, show a good correlation with liver function test; in particular they show a direct correlation with bilirubin serum levels and an inverse correlation with blood prothrombin activity. These results suggest that the VOCs breath concentration may be a direct marker of liver disease severity and, as a consequence, an important clinical parameter. In contrast, no significant correlation was found between the levels of albumin and the 12 VOCs identified. This is probably due to the little variability of serum albumin concentration in our population.

Finally, the diagnostic performance of the breath analysis was evaluated by ROC analysis. Data confirmed that monoterpenes concentration could be a good parameter to distinguish both cirrhotic patients from healthy subjects (with sensitivity and specificity of 83% and 86%) and advanced cirrhosis from early-stage cirrhosis (sensitivity and specificity of 100%). On the other hand, the N-compound seems to be able to distinguish between patients with well compensated liver cirrhosis and controls subjects whit a sensibility and specificity of 83%.

In conclusion, to the best of our knowledge, this is the first study using analysis of VOCs by direct injection mass spectrometry, and PTR-ToF-MS in particular, in the exhaled breath of cirrhotic patients. The PTR-ToF-MS breath-print of liver cirrhosis allowed to distinguish cirrhotic patients from healthy subjects and well compensated liver disease from more advanced liver stage. The breath analysis carried out with PTR-ToF-MS is a non-invasive and rapid method that allows to have a result at the time of sampling. The breath analysis can also be applied to patients who are unable to perform blood sampling and it is a tool of paramount relevance in the health service plan constantly searching for methods easy to perform and engendering high patients compliance. Our findings strongly support the availability in the near future of high throughput effective, easy, direct, and reliable method for the screening of cirrhosis.

The extensive applicability of this methodology suggest that breath analysis by PTR-ToF-MS can be a breakthrough innovative tool in diagnosis and monitoring of the progression of liver diseases.

## Supporting Information

Figure S1 **Example of PTR-TOF-MS spectra of exhaled breath.**
(TIF)

Table S1 **Markers significantly different between healthy controls (CTRL) and Child-Pugh B+C (CP-B+C) cirrhotic patients.**
(DOC)
