# Supplementary figures and images for: Rapid “Breath-Print” of Liver Cirrhosis by Proton Transfer Reaction Time-of-Flight Mass Spectrometry. A Pilot Study
Source: PLoS One. 2013 Apr 3;8(4):e59658. doi: 10.1371/journal.pone.0059658 (PMC3616040; doi:10.1371/journal.pone.0059658)

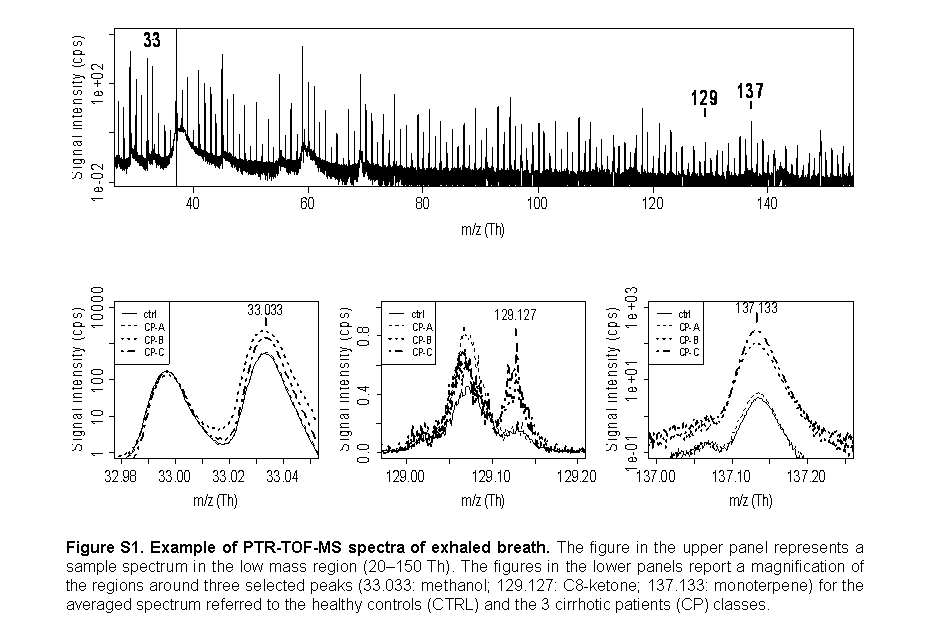

Supplement: Figure S1 — Example of PTR-TOF-MS spectra of exhaled breath. (TIF) [file pone.0059658.s001.tif]
