# Supplementary material for: Rapid “Breath-Print” of Liver Cirrhosis by Proton Transfer Reaction Time-of-Flight Mass Spectrometry. A Pilot Study
Source: PLoS One. 2013 Apr 3;8(4):e59658. doi: 10.1371/journal.pone.0059658 (PMC3616040; doi:10.1371/journal.pone.0059658)
Supplement: Table S1 — Markers significantly different between healthy controls (CTRL) and Child-Pugh B+C (CP-B+C) cirrhotic patients. (DOC) [file pone.0059658.s002.doc]

**Table S1.** Markers significantly different between healthy controls (CTRL) and Child-Pugh B+C (CP-B+C) cirrhotic patients.

| ***VOC*** | ***Concentration (ppb****_v_****)***  ***Median and Median absolute deviation*** | | ***p value*** |
| --- | --- | --- | --- |
|  | **CTRL** | **CP-B+C** |  |
| **Ketones** |  |  |  |
| 2-butanone | 2.6 ± 0.5 | 4 ± 1 | 0.001 |
| 2- or 3-pentanone | 1.06 ± 0.16 | 1.5 ± 0.4 | <0.001 |
| C8-ketone | 0.09 ± 0.01 | 0.19 ± 0.08 | <0.001 |
| C9-ketone | 0.07 ± 0.02 | 0.09 ± 0.08 | 0.028 |
| **Terpenes** |  |  |  |
| Monoterpenes | 1.3 ± 0.4 | 54 ± 52 | <0.001 |
| Terpene related | 0.38 ± 0.03 | 0.8 ± 0.3 | <0.001 |
| **S and N containing compounds** |  |  |  |
| Sulfoxide-compound | 0.06 ± 0.02 | 0.10 ± 0.03 | 0.001 |
| S-compound | 0.13 ± 0.03 | 0.06 ± 0.04 | <0.001 |
| NS-compound | 0.58 ± 0.20 | 1.4 ± 0.7 | <0.001 |
| N-compound | 0.16 ± 0.06 | 0.2 ± 0.3 | ns |
| **Alcohol** |  |  |  |
| Methanol | 279 ± 134 | 725 ± 320 | 0.008 |
| Heptadienol | 0.9 ± 0.2 | 6 ± 4 | <0.001 |
